# Supplementary material for: Exploring the prevalence of Human Papillomavirus (HPV) genotypes in PAP smear samples of women in northern region of United Arab Emirates (UAE): HPV Direct Flow CHIP system-based pilot study
Source: PLoS One. 2023 Sep 6;18(9):e0286889. doi: 10.1371/journal.pone.0286889 (PMC10482270; doi:10.1371/journal.pone.0286889)
Supplement: S1 File — Describe comparability of assessment methods if there is more than one group. (PDF) [file pone.0286889.s001.pdf]

Data sources/ measurement

8* For each variable of interest, give sources of data and details of methods of assessment (measurement). Describe comparability of assessment methods if there is more than one group

A total of 04 different age groups were concluded in this study population. Among them, 34 (32.7%) patients aged between 20-29 years, 39 (37.5%) patients fall in the 30-39 age group, the 40-49 age group included 27 (26%) patients, and 04 (3.8%) patients were the age group of 50–59-year-old and these details are given in the Table 1 & 4. All the patients were grouped in to two ethnicities: Arab and non-Arab. A total of 54 patients were Arab which accounted for 51.9%; among them 31 (49.2%) tested positive to HPV and 23 (56.1%) were negative. 50 cases (48.1%) were grouped under non-Arab; in that, 32 (50.8%) tested positive and 18 (43.9%) were negative to HPV infection.

A detailed descriptive data of the patients' age group, ethnicity, HPV positivity, single HPV genotype detection with low risk, high risk genotypes and multiple genotypes with low risk and high-risk genotypes are presented in the Table 4.

Table 1. Grading of PAP smear samples collected from women with different age groups. (Page No.10)

Table 2. Detection of HPV in different abnormal cytological and normal epithelial samples. (Page No. 10)

Table 3. Number of single, multiple low and high-risk HPV genotypes in different cytology samples. (Page No.12)

Table 4: Frequency of single, multiple low and high-risk HPV genotypes with different age group of both Arab and non-Arab study population. (Page No.13)

All the above-mentioned tables (Table 1, 2, 3 & 4) are given in the manuscript.
