# Supplementary material for: Exploring the prevalence of Human Papillomavirus (HPV) genotypes in PAP smear samples of women in northern region of United Arab Emirates (UAE): HPV Direct Flow CHIP system-based pilot study
Source: PLoS One. 2023 Sep 6;18(9):e0286889. doi: 10.1371/journal.pone.0286889 (PMC10482270; doi:10.1371/journal.pone.0286889)
Supplement: S2 File — (a) Report numbers of individuals at each stage of study—e.g., numbers potentially eligible, examined for eligibility, confirmed eligible, included in the study, completing follow-up, and analysed. (PDF) [file pone.0286889.s002.pdf]

Participants

13* **(a) Report numbers of individuals at each stage of study—eg. numbers potentially eligible, examined for eligibility, confirmed eligible, included in the study, completing follow-up, and analysed**

A total of 104 liquid-based cervical cytology samples received from various hospitals of Northern Emirates UAE to the Department of Pathology at Thumbay laboratory, Thumbay University Hospital, Ajman, UAE to diagnose the cervical abnormalities and detect the HPV positivity and their genotypes. The liquid based cervical cytology samples were obtained from women aged 20-59 years attending the Gynaecology out-patient department of Thumbay University Hospital and other hospitals of Northern Emirates of UAE. Upon arrival, the samples were immediately processed for cytological examination and detection of HPV positivity and their genotypes as per the laboratory protocol. All the samples were used in this study and their HPV positive and negative results were documented.
