# Supplementary material for: Exploring the prevalence of Human Papillomavirus (HPV) genotypes in PAP smear samples of women in northern region of United Arab Emirates (UAE): HPV Direct Flow CHIP system-based pilot study
Source: PLoS One. 2023 Sep 6;18(9):e0286889. doi: 10.1371/journal.pone.0286889 (PMC10482270; doi:10.1371/journal.pone.0286889)
Supplement: S4 File — (PDF) [file pone.0286889.s004.pdf]

Outcome data

15* Cross-sectional study—Report numbers of outcome events or summary measures

A total of 104 liquid based cervical smears samples were processed for the routine cytopathological examination to identify and differentiate the morphological changes of the PAP smear samples. Based on Bethesda system 2014, the samples were graded as ASCUS (12.5%; n=13), LSIL (6.7%; n=07), HSIL (0.96%; n=1) & ASC-H (0.96%; n=1) and normal epithelial cells (78.84%; n=82) and their details are presented in Table 1, and Fig 1 & 2. Out of 104 PAP smear samples, 63 were positive and 41 were negative to HPV infection and it is mentioned in Table 2.

In total, 112 HPV genotype were encountered in all the 63 positive samples (18 AC & 45 NC). In the HPV positive ASCUS samples, HR-HPV 16 were detected in 3 samples and LR genotypes 11 & 70 were detected in 2 samples. In the LSIL samples, the LR-HPV 6 & 16 were detected in 2 samples and in the case of HSIL and ASC-H cytology, the LR-HPV 62/81, 35 & HR-HPV 67,16 were detected. In normal cytology, the genotype HPV6, 11, 45, 68, 70, 82 were detected in 11, 8, 7, 3 and 2 samples respectively and the HPV31 and 16 were encountered in 4 different samples. Number of single and multiple, low and high-risk genotypes detected in different cytology samples are given in Table 3 and in Fig 3.

A total of 04 different age groups were concluded in this study population. Among them, 34 (32.7%) patients aged between 20-29 years, 39 (37.5%) were fall in the 30-39 age group, the 40-49 age group included 27 (26%) patients, and 04 (3.8%) were the age group of 50–59-year-old and the details are given in the Table 1 & 4. All the patients were grouped in to two ethnicities: Arab and non-Arab.

A total of 54 patients were Arab which accounted for 51.9%; among them 31 (49.2%) tested positive to HPV and 23 (56.1%) were negative. 50 cases (48.1%) were grouped under non-Arab; in that, 32 (50.8%) tested positive and 18 (43.9%) were negative to HPV infection. Detailed descriptive data of results is presented in the Table 4.

In the age group of 20 – 29-year-old Arab ethnicity, 10 HPV genotypes (HPV11; n=2, 42, 44/45, 31/68, 11/67, 16, 45, 54, 58) were detected, in the age group of 30 – 39 year old patient, 22 genotypes (HPV11;n=2, 6;n=5, 16;n=2, 51, 43, 67;n=2, 70, 52/86, 45;n=3, 59, 82, 61, 54) were observed and the HPV6 was the common genotype. There are 21 genotypes (HPV6;n=3, 70,

11;n=2, 18, 45, 68, 62/81;n=2, 43, 42, 51, 16;n=2, 82, 61, 31, 51, 67) were detected in the age group of 40 – 49-year-old of the same ethnicity and these details are given in Fig 3, 4 & 5.

In the non-Arab ethnicity, 25 genotypes (HPV11, 84, 58;n=2, 16;n=4, 73, 45;n=3, 67;n=2, 61, 39, 62/81;n=2, 31, 54, 70, 18, 33;n=2, 68) were detected in the age group of 20 – 29-years old and the HR-HPV16 was predominant. The HR-HPV18 & 16 were identified together in one sample along with HPV54, 70 and 33. In the age group of 30 – 39-year-old, 24 genotypes (HPV6;n= 4, 40, 59, 66, 70;n=2, 43, 62/81, 56, 68;n=3, 31, 44/55, 54, 45, 67, 16, 84, 39, 58) were detected and the HPV6 were commonly identified. The HPV16 was detected in one sample along with HPV67. 10 HPV genotypes (HPV82, 31, 62/81;n=2, 6;n=2, 42, 11, 39, 35) were detected in the age group of 40–49-year-old patient group and the genotype HPV11 was detected in one sample of the age group 50 – 59-year-old of the same ethnicity and these details are given in the Fig 3, 4 & 5 and Table 4.

Among the 63 HPV positive samples, 39.68% (n=25) samples were infected by single HPV genotypes, of which 19.04% were infected by only low-risk genotypes and whereas high-risk single genotypes detected in 20.63% samples. Multiple genotypes were detected in 60.31% (n=38) samples; in that, 19.04% were infected with low-risk genotypes, 7.93% were with high-risk genotypes and 33.33% (n=21) samples were infected by multiple genotypes of both low and high-risk types and these details are given in the Table 5, 6 and Fig 5. Prevalence of the low and high-risk genotypes HPV6 (13.46%), HPV11 (9.61%), HPV62/81 (7.69%), HPV16 (9.61%) and HPV45 (7.69%) were moderately high in this finding.

***ASCUS** - Atypical squamous cells of undetermined significance, **HSIL** - High grade squamous intraepithelial lesion, **LSIL** - Low grade squamous intraepithelial lesion, **ASC-H** - Atypical squamous cells cannot rule out high grade squamous intraepithelial cells.

* HR-HPV – High-risk Human Papillomavirus, LR-HPV – Low-risk Human Papillomavirus
