# Supplementary material for: Exploring the prevalence of Human Papillomavirus (HPV) genotypes in PAP smear samples of women in northern region of United Arab Emirates (UAE): HPV Direct Flow CHIP system-based pilot study
Source: PLoS One. 2023 Sep 6;18(9):e0286889. doi: 10.1371/journal.pone.0286889 (PMC10482270; doi:10.1371/journal.pone.0286889)
Supplement: S5 File — (PDF) [file pone.0286889.s005.pdf]

Interpretation

20. Cautious overall interpretation of results considering objectives, limitations, multiplicity of analyses, results from similar studies, and other relevant evidence

The present study aimed to explore the prevalence of low and high-risk HPV genotypes in PAP smear samples of women in northern region of the UAE using HPV direct flow CHIP method. Out of 104 PAP smear samples, 21.16% of the samples showed abnormal cytology (ASCUS - 12.5%, LSIL - 6.7%, HSIL & ASC-H - 0.96%) that is precancerous type and 78.84% appeared normal cytology. The abnormal cervical cytology rate has increased nearly 60% in recent years in the UAE when compared with previous studies reported in 2019 [19], in 2015 [20, 21] and in 2006 [12]. The current prevalence of cervical cytological abnormalities was 21.15% and it is being higher than the reports from the neighboring country Oman (6.92% & 3.5%) [15, 22] and lower than the reports from Iran [25%] [24]. The current rate was almost nearer to the reports which were from USA [19.6%] & India [19.6%] in 2022 [25, 10]. The current rate was lesser than the reports which were published in 2016 by Sign [46.8%] in UAE [26].

Prevalence of HPV in this study was 60.58%, this was lesser than a previous study (88%) was conducted in 2018 in UAE [16] and this lesser rate has been shown a significant positive awareness of the HPV infection and vaccination among the population. But in the year 2015 & 2017 [20, 28], the HPV prevalence rate in the UAE was 17.9% and 44.38%, these lower rate might be the reason for lack of cutting-edge techniques like flow through reverse hybridization had not been available to detect the different LR and HR genotypes. The prevalence of HPV infection in the abnormal and normal cytology samples was 27.83% and 72.17% respectively.

In the age group of 20 – 29-year-old study population, HPV16 & 45, 11, 58 & 67 were the frequently identified. In this age group, HPV11 (16.7%) was the predominant in Arab patients and HPV16 (22.2%) was in non-Arab group. In the age group of 30-39-year-old patient, the most frequently identified genotypes were HPV6, 45 & 16. Like this, in the age group of 40 – 49, HPV6, 62/81, 11 were the common genotypes. None of the HPVs were detected in the 50 – 59-year-old study group except HPV 11. Across all the age group of Arab patients, the HPV6, 11, 45, 16 & HPV67 were the most commonly detectable genotypes and in the non-Arab

patients, the HPV6, 62/81, 16 and 45 were the commonest genotypes. However, the HR-HPV18 was identified in both group but the positivity rate was 1.8% and like this, low positivity rate of HPV18 was detected in the Western Iranian study group [24].

The LR-HPV 6 was the most predominant genotype among all the identified HPVs and then HPV16, 45, 11, 62/81 & 67 were the next detectable genotypes. Like this in the Iran study group, the HPV6 was the predominant type [24]. But in other previous studies which were conducted in UAE [13], China [29], India [10], Dutch-Caribbean Island [30], Saudi Arabia [31] and Western Mexico [33], the HPV 16 was the predominant genotype. But in the Oman study population, LR-HPV54 was the predominant genotype followed by HPV82, 42, 68 & 44 [15].

In our study, the higher frequency of HPV infection rate was encountered in the age group of 20 – 49 years old of both study group. Similarly, a study conducted in Abu Dhabi, UAE in 2018, stated that the maximum infection rate was observed in the age group between 24-54 years old women [13] and in the year 2016 & 2017, the studies which were conducted in the UAE, mentioned that the maximum HPV infection rate was observed in women over 30-year-old [20, 289]. In Hefei province of China, the maximum infection rate was detected in the age group of 31-50-year-old women [29] and in Western Iran the maximum HPV infection rate was determined in the age group of 31-40-years-old women [24] and in Saudi Arabia, rate was identified in between the age group 30-50-year-old women [31]. The highest HPV positivity rate (85%) was detected in women aged 36–55-year-old from Western Mexico [32]. But some previous studies stated that there was no association between HPV infection and the stratified age groups [≤25, 25-34. 35-44 and ≥45 years] [33]. However, in the developed counties the prevalence of HPV was peak in young women and lowered after 35 years of age [5] and other studies mentioned that there is a second peak in the postmenopausal age groups in some countries [34, 35].

The present study showed that 60.58% of women with cervical infection and/or inflammation had HPV positivity. Among them the high-risk HPVs rate was 14.8%, low-risk genotypes rate was 23.08% and mixed LR and HR genotypes infection rate was 20.3%. Whereas, in Brazil 88.4% of high-risk genotypes was reported and it was significantly higher [36]. The study provided age-specific HPV infection rate among women of Arab and non-Arab cohort. The women aged 20 - 49 years had the highest infection rate [60.29%]. The study found that the prevalence of HPV infection is higher in the younger age group and declined

gradually in the older age group. But in northern Henan Province of China, the rate of HPV infection was higher in 60-year-old women and lower in the middle age group [37].

Conclusion

The current findings confirm UAE to have a slightly high HPV prevalence. According to the study, HPV 6, 11, 16 and 62/81 were the most common HPV infections in the women between 21 and 59 years old and much less prevalence of HPV 18 was found. A moderate increase than expected incidence of HPV45 and 62/81 were detected. Co-infection with multiple low and high-risk genotypes is present in 20.2% of cases. Among them, HPV6 was most common followed by HPV62/81 and HPV16. 43.27% of normal epithelial PAP smears were positive to HPV infections with single and multiple of both low and high-risk genotypes. It is evident that symptomatic women even if having normal epithelial PAP smear should be screened for HPV genotyping and followed up with periodic PAP smears for detecting any cytological changes of cervix. The study highlights the importance of molecular techniques to emphasize cervical screening triage. A large population-based study is needed to determine the most prominent genotypes and develop new vaccines to reduce the burden of cervical infection.

Limitations of the study

As of all other studies this study also reported few limitations as follow. The study samples received from various hospitals, clinics and Thumbay hospitals located in and around the northern emirates of UAE (Sharjah, Ajman, Umm-Al-Quwain, and Fujairah) and not received the samples from other emirates (Dubai, Abu Dhabi & Ras-Al-Khaimah). In this HPV Direct Flow CHIP method, the mentioned genotypes HPV62/81, 31/68 & 44/55 could not be able to consider as a single or mixed genotype.
