# Supplementary material for: Exploring the prevalence of Human Papillomavirus (HPV) genotypes in PAP smear samples of women in northern region of United Arab Emirates (UAE): HPV Direct Flow CHIP system-based pilot study
Source: PLoS One. 2023 Sep 6;18(9):e0286889. doi: 10.1371/journal.pone.0286889 (PMC10482270; doi:10.1371/journal.pone.0286889)
Supplement: Attachment — Submitted filename: Response to reviewer.docx [file pone.0286889.s006.docx]

**Response to Reviewers**

**From**

**Dr. NAZEERULLAH RAHAMATHULLAH B.Sc, M.Sc, Ph.D**

Assistant Professor of Microbiology & Immunology

Department of Biomedical Sciences, College of Medicine

Gulf Medical University,

Ajman, United Arab Emirates.

**To**

**THE EDITOR-IN-CHIEF**

PLOS ONE

San Francisco, CA 94111

United States.

**Sub**: Response to Reviewers on the Manuscript entitled *“Exploring the prevalence of Human*

*Papillomavirus (HPV) genotypes in PAP smear samples of women in northern region of United Arab Emirates (UAE): HPV Direct Flow CHIP system-based pilot study".* [PONE-D-23-11894]

**Dear Sir/Madam,**

Greetings!

First, I would like to thanks to editorial manager & academic editor to review my manuscript. Sir, I have revised the manuscript according to the academic editor and reviewer(s) comments. The following comments mentioned by the reviewers are noticed and corrections are done accordingly in the revised manuscript.

**Reviewer # 1**

**Abstract**

**Comment 1:** Conclusion should summarize the key learning from the study and implications for practice, policy, and research. The results provided in lines 40 to 45 should be moved to Results section.

**Ans:** The above-mentioned comments were responded in the conclusion and the changes are highlighted and the results provided in lines 40 to 45 are moved to the results section.

**2.2 Subjects**

**Comment 2**: Line 107 - either describe the laboratory protocol or provide a reference if the protocols are a standard protocol.

**Ans:** As per the comment, the reference is provided in that section (**line 119**) and highlighted.

**2.7 Statistical Analysis**

**Comment 3:** Line 147, it is sufficient to say "data were analyzed", please delete "and statistically evaluated"

**Ans:** The suggestion is added in the line – **159 to 161**.

**3. Results**

**Comment 4:** Please remove leading zeros before integers on lines 156, 173, and 175.

**Ans:** The leading zeros are removed before integers on the lines **167, 189, 191** and highlighted in the revised manuscript.

**Comment 5**: Line 179, start the sentence using "Fifty cases" and not "50 cases".

**Ans:** The above-mentioned issue instead of “50 cases”, the word **“Fifty cases**” is added in the line **195**.

**Comment 6**: Table 2 (page 10), Pearson's correlation analysis not described in the Methods section. Please describe all analysis in the Methods section.

**Ans:** By mistake, the Pearson’s correlation analysis was mistakenly mentioned in the table 2. Originally, the Pearson’s Chi-square test was performed in tables 2, 3 & 6 with *p*-value ˂ 0.05 considered as statistically significant. It is mentioned in the revised manuscript on the line 159 to 161 and in the table 2, 3 & 6.

**References**

**Comment 7:** Lines 390 to 391 (reference 3), please use proper referencing style for a webpage showing date accessed. Recommend using a Reference manager if the references were done manually for consistency.

**Ans:** The proper referencing style is used (**3.** World Health Organization. Cervical cancer. 2022. <https://www.who.int/news-room/fact-sheets/detail/cervical-cancer>.) updated in the attached revised copy and highlighted in the line – **404 to 405**.

**Comment 8:** A general comment,

The manuscript will benefit from scientific editing by a native English speaker. For example, the sentence on lines 280 to 283 is unclear. Also, the phrase "Like this" was used too frequently (lines 284, 291, 298 and 301). The language should be varied.

**Ans:** The above-mentioned general comments are noticed and the corrections are done accordingly in the discussion part (from the line **279 to 319)**.

**Reviewer# 2**

I am recommending the article on Exploring the prevalence of Human Papillomavirus (HPV) genotypes in PAP smear samples of women in northern region of United Arab Emirates (UAE): HPV Direct Flow CHIP system-based pilot study for publication. The research is novel.

**Reviewer #3:**

**Comment 9**: Although the manuscript is technically sound, the analysis well done and presented in an intelligent fashion, the writing standard should be improved on, to make statements in the result and discussion flow for easy understand.

For that, the following points need to be taken into consideration:

**INTRODUCTION:** there should be additional scholarly evidence/reasons for the conduct the research.

**Ans:** The above comment is considered and the reasons for the conduct the research is added in the end of the introduction (from the line **86 to 92**).

**METHOD:**

I suggest:

**Comment 10:** 1. There should be sections explaining;

**a. Data Collection**

**Ans:** The above suggestion is included in the method section of the revised manuscript (line **108 to111**) and highlighted.

**b. study design**

**Ans:** The above suggestion is incorporated and highlighted in the revised manuscript (from the line 103 to 107).

**Comment 11:** 2. Study Populations should be used instead of "Subjects".

**Ans:** The suggestion is added in the revised manuscript (line 112).

**Comment 12**: 3. There should be a clear explanation of what was done with the sturdy samples to explain the variation in some reported parameters e.g. the cytology examination result repeated, why is the difference between the first and the second reported cytology result in section 3.1 and 3.2.

**Ans:** The above-mentioned comment is noticed, and the corrections are done in the result section of the revised manuscript.

3.1. **Study samples and Cytological assessment** – This title has been updated in to “**Cytological Examination**”. In this heading the different morphological changes of the cervical PAP smear samples’ results are discussed. How many ASCUS, LSIL, HSIL and ASC-H are identified. How many PAP smear samples have shown normal epithelia even the woman has cervical symptoms, and their details are given in the table 1 and figure 1 & 2.
     **3.2. HPV genotype detection** – This title is updated into “**HPV detection and genotyping”** – In this heading, results of how many ASCUS, LSIL, HSIL, ASC-H and normal epithelia are positive to HPV infection, type of the genotypes such as low and high-risk and the predominant HPV genotypes in the samples, and their details are given in the table 2 & 3 and figure 3. The positivity of the HPV infection was done by multiplex Polymerase chain reaction and their genotype was done by flow-through reverse hybridization using HPV direct flow chip kit and it is mentioned in the method section – Flow through reverse hybridization (line 144 – 157).

**Comment 13:** Look at lines 164, 165 and 166 and rephrase the sentence for better understanding.

**Ans:** The above comment is noticed and rephrased the lines (164, 165 and 166) 173 to 174 & 176 to 182 and highlighted in the revised copy. These sentences are rephrased for better understanding.

**RESULT**
I suggest
**Comment 14:** 1. You should re-write the different sections of the result to make the findings simple to understand.

**Ans:** Yes, the result section has been written and rephrased to make the findings simple to understand.

**Comment 15:** 2. Look again at the tables to make them simpler,

**Ans:** The tables 1, 2, 3, 4, 5 & 6 are simplified and highlighted in the simplified areas.

**Comment 16:** 3. Review the need to use superscript a,b,c,d,e, etc for "n".

**Ans**: All the superscripts mentioned in the tables (1,2,3,4,5 & 6) are removed, simplified, and highlighted in the revised manuscript.

**DISCUSSION**

**Comment 17:** Discuss the result only, no need to repeat what was already mentioned in the result section. e.g. what is in lines 262 and 263 is a repeat of a statement that is already in the result section.

**Ans:** The above-mentioned comment is noticed, and the corrections are done accordingly at the lines 278 -286, 293 -294, 295 -300, 302, 306, 313, 317 -318, 334 in the revised manuscript.

**CONCLUSION**

**Comment 18**: Review the conclusion to distinguish between finding from the study and recommendation based on the findings. e.g. the statement in line 340, 341, and 342 contains what looks like a finding from the study but ended up with a recommendation.

**Ans:** As per the comment, the conclusion is revised in the updated manuscript and highlighted (Lines 355 to 360).

**Reference section**

The references 27, 33 & 37 are arranged as per the journal’s reference style and highlighted in the revised manuscript.
